# Students as Community Vaccinators: Implementation of A Service-Learning COVID-19 Vaccination Program

**DOI:** 10.3390/vaccines10071058

**Published:** 2022-06-30

**Authors:** Andrew R. Griswold, Julia Klein, Neville Dusaj, Jeff Zhu, Allegra Keeler, Erika L. Abramson, Dana Gurvitch

**Affiliations:** 1 Weill Cornell/Rockefeller/Sloan Kettering Tri-Institutional MD-PhD Program, New York, NY 10065, USA; ang2055@med.cornell.edu (A.R.G.); ned2010@med.cornell.edu (N.D.); 2 Weill Cornell Medicine, New York, NY 10065, USA; jik4001@med.cornell.edu; 3 Clinical & Translational Science Center, Weill Cornell Medicine, New York, NY 10065, USA; jez2003@med.cornell.edu (J.Z.); alk4016@med.cornell.edu (A.K.); 4 Joan and Sanford I. Weill Department of Medicine, Weill Cornell Medicine, New York, NY 10065, USA; err9009@med.cornell.edu; 5 Department of Anesthesiology, Weill Cornell Medicine, New York, NY 10065, USA

**Keywords:** vaccination, COVID-19, vaccine, service-learning, medical education, clinical skills

## Abstract

While the COVID-19 pandemic has caused major educational disruptions, it has also catalyzed innovation in service-learning as a real-time response to pandemic-related problems. The limited number of qualified providers was primed to restrict SARS-CoV-2 vaccination efforts. Thus, New York State temporarily allowed healthcare professional trainees to vaccinate, enabling medical students to support an overwhelmed healthcare system and contribute to the public health crisis. Here, we describe a service-learning vaccination program directed towards underserved communities. A faculty-led curriculum prepared medical students to communicate with patients about COVID-19 vaccines and to administer intramuscular injections. Qualified students were deployed to public vaccination clinics located in under-served neighborhoods in collaboration with an established community partner. Throughout the program, 128 students worked at 103 local events, helping to administer 26,889 vaccine doses. Analysis of a retrospective survey administered to participants revealed the program taught fundamental clinical skills and was a transformative service-learning experience. As new virus variants emerge and nations battle recurrent waves of infection, the need for effective vaccination plans continues to grow. The program described here offers a novel framework that academic medical centers could adapt to increase vaccine access in their local community and provide students with a uniquely meaningful educational experience.

## 1. Introduction

Service learning is an experiential educational approach that combines community engagement with academic coursework and personal reflection [1,2]. It is a core component of medical training and required for institutional accreditation by the Liaison Committee on Medical Education (LCME) [3,4]. Over the past two-years, the COVID-19 pandemic has disrupted the delivery of medical education and forced the role of medical students to evolve. Nevertheless, medical students have found ways to contribute to the overwhelmed health care system and serve their community in these uncertain times. While many initiatives occurred in non-patient facing roles [5,6,7,8,9], some service-learning experiences incorporated direct patient contact. Most notably, perhaps, was the accelerated graduation and deployment of senior medical students as new physicians [10,11]. Students have also screened patients for COVID-19 [12], provided telemedicine support [13], worked at patient call centers [14], and facilitated the reopening of student run free-clinics [15]. Collectively these examples show that although medical students are generally accepted to be non-essential workers [16]; they do represent a large pool of untapped providers with tremendous potential.

The shortage of qualified healthcare professionals capable of administering vaccinations at the scale required early in the pandemic presented a unique service-learning opportunity to medical students with historic precedent. During the great influenza pandemic of 1918, American medical students assisted with the mass inoculation program [17]. Indeed, on 8 January 2021, New York expanded executive order 202.9 which enabled professional students to become eligible vaccinators given they met the established standards. Despite the guidance provided by this executive action, the development and execution of student programs remained the responsibility of academic institutions. We were particularly interested in how to create a sustainable program that serviced underrepresented communities while being a value-added experience for medical student volunteers. However, there was a dearth of scholarship about student-run vaccination clinics and no literature about a vaccination program of the scale and duration envisioned for COVID-19. Here we describe the design, implementation, and evaluation of our institution’s first of its kind student vaccinator program.

## 2. Materials and Methods

### 2.1. Program Development

Following the executive order enabling students to serve as vaccinators, Weill Cornell Medicine began training students as volunteer vaccinators to staff community vaccination sites organized by the Community Healthcare Network (CHN), a federally qualified health center. Guidelines for student training were set by the NYS Department of Health (DOH) to include completion of at least one year of clinical training, vaccine specific online training modules, an in-person training session, and BCLS certification. The online training modules included specific education about counseling and administration of the Pfizer-BioNTech COVID-19 vaccine (Comirnaty), the Moderna COVID-19 vaccine (Spikevax), and the Johnson and Johnson COVID-19 vaccine consistent with Emergency Use Authorization (EUA) and Centers for Disease Control and Prevention (CDC) guidelines. Students who had completed the full foundational curriculum in addition to at least one clerkship were eligible for training. Student volunteers were required to be fully vaccinated or willing to take both vaccine doses at associated vaccine events. Students who did not meet the clinical curricular requirements and/or were not vaccinated were excluded from training and volunteering.

A skills checklist for the in-person skills demonstration was provided by the NYC.GOV website (https://www.immunize.org/catg.d/p7010.pdf (accessed on 2 February 2021)). In-person skills training session were held in an Objective Structured Clinical Examination (OSCE) format familiar to medical students under supervision of an anesthesiology resident and/or attending physician. All NYS guidelines for Personal Protective Equipment (PPE) were strictly adhered to and measures were discussed regarding managing adverse outcomes. During the OSCE-style sessions, students were supervised conducting a complete mock COVID-19 vaccination encounter including patient education, vaccine preparation, and immunization administration of a saline intramuscular (IM) injection on a fellow classmate. Upon successful completion of all training components, students were added to the certified roster and became eligible to volunteer at vaccination events.

### 2.2. Vaccination Events

**Figure 1:**
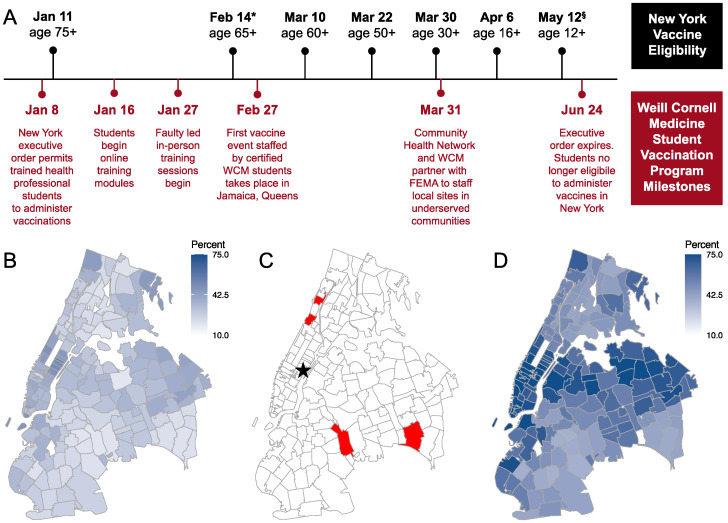
Timeline and map of student vaccination program in 2021 (**A**) New York State COVID-19 vaccine eligibility dates (top, black) superimposed with summary of key events of WCM vaccination program (bottom, red). Note timeline not drawn to scale. * Vaccine eligibility also extended to people with underlying medical conditions on February 14. ^§^ People ages 12–15 were exclusively eligible for the Pfizer-BioNTech COVID-19 vaccine. (**B**) Heatmap of NYC vaccination status, at least one dose by 26 March 2021. (**C**) Locations of WCM vaccination program sites (red) and Weill Cornell Medicine (star). (**D**) Heatmap of NYC vaccination status at least one dose, by 1 July 2021, as colored in (**B**).

Vaccination events occurred from February to July 2021. Patient eligibility was in accordance with NYS guidelines (Figure 1A). Each event had medical oversight by either an attending physician or nurse practitioner. Students were responsible for reconstituting vaccine doses, administering vaccinations, counseling patients regarding vaccine expectations, and appropriately documenting vaccinations in the New York state database. All tasks were performed jointly by medical students, nurses from our partner clinic, and attending physicians and nurse practitioners. Students were encouraged to seek assistance from a senior provider if they had questions or required help counseling patients (most commonly pregnant women, children, and patients with chronic disease). Data on vaccine recipients between were collected by Community Health Network. Demographic breakdowns of patients were calculated for Age, Sex at Birth, and Race/Ethnicity.

Vaccination events took place throughout NYC at transient (pop-up) and stable (Federal Emergency Management Agency (FEMA)-funded) sites. The permanent locations were intentionally established in under-served and under-represented communities: Church of God in Brooklyn, Fort Washington Collegiate Church in Washington Heights, New Jerusalem Worship Center in Jamaica Queens and Convent Avenue Baptist Church in Harlem (Figure 1B–D). Transportation was provided for student volunteers. These sites were open on weeknights and weekends to minimizes barriers to access for working individuals. The Pfizer-BioNTech COVID-19 vaccine (Comirnaty), the Moderna COVID-19 vaccine (Spikevax), and/or the Johnson and Johnson COVID-19 vaccine was offered based on local availability. Community partners provided critical support including check-in and scheduling to translation assistance and custodial work. FEMA and NY Department of Health (NYDOH) delivered vital backing—Ensuring an uninterrupted supply of vaccines, creating a digital infrastructure for tracking patient data, and providing the funds for much of this program.

### 2.3. Retrospective Survey Development and Administration

The program was evaluated with a retrospective survey. Questions for medical student vaccinators were developed to address the following broad categories: quantify degree of involvement in the vaccination program, safety, skills development (hands-on and communication with patients), areas for improvement, and personal reflections. The survey was administered via RedCap [18] and included quantitative questions about training and open-ended questions on value of experience. A copy of the survey can be found in Supplementary File S1. Students who participated in at least one event were provided with anonymous online survey link via their Weill Cornell Medicine (WCM) email address (or personal email addresses for M4 students, all of whom graduated prior to distribution). Completion of the survey was optional.

## 3. Results

Our program was unique in its ability to quickly train vaccinators, place students at vaccination sites and collect data regarding this service-learning opportunity, which became a unique curricular experience for Weill Cornell Medicine (WCM) students that may serve as a model for similar service-learning efforts. The ever-changing landscape of State and Federal guidelines temporally guided many program milestones (Figure 1A). For example, within one month of the January executive order, WCM students had completed online modules and attended faculty-directed training sessions as described above. The first student-staffed vaccination event on February 27, occurred during a surge of vaccine demand corresponding to expansion of vaccine eligibility for adults 65 years of age and older and people with underlying medical conditions. At this first event, students helped inoculate 176 patients with no major adverse events, providing proof of principle that the student vaccination program was safe and effective.

### 3.1. Student Participants

Based on the inclusion criteria, 324 WCM students were eligible for vaccine training. Of these, 128 completed training and became certified vaccinators (40%). These students were from both MD and MD-PhD programs and tended to be in the later years of training (Table 1). 80 students (63% of certified) participated in at least one or more vaccination event.

### 3.2. Patient Demographics

Students helped to inoculate 14,293 individuals with 26,889 vaccine doses (Table 2). Patients were relatively equally distributed in sex at birth, with slightly more females (7145) than males (6822). The majority of the patients (9237) were younger than 45 in age. While this is contrary to national trends that elder individuals are more likely to be vaccinated [19], it likely stems from the timing of when recurring FEMA sites were established (March 31). For example, individuals ages 30–49 only become eligible for vaccines on March 30, whereas older people had been eligible for vaccines for up to 3 months (Figure 1A). Of those inoculated, 30% of patients identified as Black, 39% of patients identified as Hispanic, and 10% of patients reached by this program identified as White.

### 3.3. Evaluation of the Vaccination Program

The effect of the program as a service-learning opportunity for student participants was evaluated through a retrospective survey (Supplementary file). Fifty students (50/80, 63% response rate) completed the optional post-volunteer survey. A summary of the results as well as representative excerpts are shown in Table 3 and Table 4. Prior to training, the percent of students feeling comfortable administering IM injections was 2%, which increased to 76% after training. All students indicated that they felt adequately prepared to participate after training. One hundred percent of students indicated they were comfortable talking to patients about the COVID-19 vaccine and administering IM injections after volunteering. Similarly, the percent of students feeling comfortable talking to patients about the COVID-19 vaccine increased from 30% prior to volunteering to 100% after volunteering.

Inspection of qualitative responses indicated that participation in the vaccination program was an opportunity for professional growth. When asked why they volunteered, students indicated desires to work with vulnerable communities, reduce disparity in vaccine access, feeling a sense of civic responsibility to help with pandemic efforts and wanting to help pandemic efforts in a direct way. When describing how volunteering as a vaccinator contributed to their professional development, student responses ranged from improving communication with diverse patient populations to better understanding how large-scale public health efforts are coordinated an executed. Finally, when asked how this experience contributed to their medical education students gave many reasons, including fostering an interest in public health and gaining confidence and experience functioning as an independent provider.

## 4. Discussion

Here we discuss the development, implementation, and evaluation of an innovative service-learning COVID-19 vaccination program. While there is growing body of literature about medical student contributions to the COVID-19 response [5,6,7,8,10,12,15,20,21,22,23,24,25,26,27,28,29,30], our work is the largest student-lead, community-based vaccination program described to date. It reveals that medical students are effective front-line vaccinators during a global pandemic and can attain a valuable learning experience from this service.

Our vaccination program met its primary goals, to expand access to the COVID-19 vaccine and to provide a valuable service-learning experience for medical students. Our program evaluation survey results indicate that the training provided was well received. Training notably increased the percent of students feeling comfortable administering IM injections, with all respondents noting that they felt adequately prepared to participate. We also observed a notable increase in students’ comfort counseling patients on the COVID-19 vaccination before and after volunteering. Together these findings demonstrate that this service-learning experience provided educational value and boosted clinical confidence, solidifying both comfort with clinical skills and patient counseling. Qualitative survey responses echoed these findings. In fact, multiple students indicated that this was the most meaningful part of their medical education to date.

Crucial to the success of our program were the three key-stakeholder groups—Medical students, community partners and the government—Each of whom made unique contributions to the program. Medical students, eager to volunteer and altruistic with their motives, formed the backbone of the program workforce by increasing the number of eligible vaccinators. They were trained providers whose competency was readily identified by supervising faculty. Once trained, students could help administer vaccine doses at both their home medical institutions’ clinical locations, as well as partner locations throughout their community. Collaborating with established community partners meant efforts to be directed towards delivering care to vulnerable communities in need of vaccination infrastructure without extensive relationship building. Finally, government bodies provided vital support by guaranteeing a reliable supply of vaccines, passing policies which enabled students to become qualified vaccinators and providing key financial support required to sustain a long-term program. While the collaborative approach was overwhelming positive, one limitation was that a third-party managed the digital infrastructure tracking vaccines. As such, we were unable to retrospectively analyze vaccine data based on the provider (i.e., medical student, nurse, physician associate, or physician). Nevertheless, we believe that this three-part model integrating students, community partners and government agencies, can be utilized to enhance community-outreach programs at our institution and can be emulated for future service-based learning programs at medical schools across the country.

Given the emergence of new SARS-CoV-2 variants (BA.4, BA.5, BA.2—Omicron subvariants, B.1.1.529—Omicron, B.1.617.2—Delta) and evolving nature of the COVID-19 pandemic the need for COVID-19 vaccinations remains high. Inoculations are available to children younger than 18, booster shots are available to most adults, and at least 20% of the American population eligible to receive the vaccine has yet to receive a single dose. As the Public Readiness and Emergency Preparedness (PREP) Act continues to allow medical students to serve as vaccinators, institutions around the country have the potential to help fulfill these needs, which we anticipate will continue to grow. Internationally, there is tremendous need for augmented vaccine distribution. This is particularly the case in developing nations, many of which have immunized less than half their population [31]. Nevertheless, the majority of COVID-19 student vaccination efforts have occurred in wealthy countries such as the United States and Europe [32].

Here, we provide a skills-based, checklist-guided approach to ensure competency amongst medical student that can be easily adapted and implemented by medical schools. While this framework was designed for medical students, we anticipate it could also benefit trainees in other allied healthcare professions, many of whom often participate in vaccine distribution [33]. There is precedent for nursing, pharmacy and medical students providing vaccines in the United States. Most literature revolves around influenza vaccines [34]. For example, pharmacy students in Georgia implemented a successful mobile influenza vaccination effort, which immunized 1303 patients [35]. Similarly, a large interprofessional student healthcare workforce provided influenza vaccines to marginalized populations in Nashville, Tennessee [36]. Likewise, a team of medical, pharmacy, and nursing students in Illinois augmented influenza vaccine rates among economically disadvantaged individuals [37]. There are also examples of medical students administering multiple childhood immunizations (including Hepatitis, Human Papilloma Virus, and Tetanus Diphtheria acellular Pertussis) to middle school students in Michigan [38]. These efforts utilized similar strategies outlined in our COVID-19 vaccination program. Notably they employed thoughtful education, training, and skills development for students, along with adequate supervision by a senior healthcare provider at vaccination events. Collectively these works show that students from a variety of healthcare professions can likely enhance vaccine distribution with proven effectiveness. We hope our meaningful and impactful service-learning model will be readily adopted.

## 5. Conclusions

Medical students are uniquely positioned to aid in the distribution of vaccines during a pandemic such as COVID-19. In doing so, students are able to benefit under-resourced communities and increase vaccine availability, while also having the opportunity for service-learning which improves proficiency in IM injections and patient counseling. Collaboration amongst various stakeholders is key to the success of such an initiative. Medical school faculty and residents provide training to volunteer medical students, community partners identify communities in need of assistance, and government bodies provide vaccines and permit students to administer care in this capacity. As the COVID-19 pandemic evolves, and the need for additional vaccination remains ever-present, other academic medical institutions can rapidly adapt this service-learning model which enhances student education and improves community health.

## Figures and Tables

**Table 1:** Demographics of students certified as vaccinators.

Student Vaccinator Characteristic	% (N = 128)
Program	
MD	68 (87)
MD-PhD	32 (41)
Class Year	
2	13 (16)
3	34 (44)
4+	53 (68)

**Table 2:** Demographics of patients vaccinated through the Program.

Patient Demographic	% (N = 14,293)
Age	
<18	17 (2451)
18–24	12 (1683)
25–34	18 (2548)
35–44	18 (2555)
45–54	15 (2147)
55–64	13 (1796)
65+	7 (1057)
Sex at Birth	
Female	50 (7145)
Male	48 (6822)
Race/Ethnicity	
White	10 (1390)
Black	30 (4345)
Asian	10 (1365)
Hispanic	39 (5557)
Other	9 (1287)

**Table 3:** Summary of student evaluations about vaccination experience.

Program Evaluation Question	% (N = 50)
Did you feel you had adequate training and preparation to participate?	
Yes	100% (49)
No	0% (0)
Did you feel protected wearing appropriate PPE and adhering to NYS guidelines?	
Yes	100% (50)
No	0% (0)
How comfortable did you feel with IM injections prior to training?	
Not at all or somewhat comfortable	98% (49)
Comfortable or very comfortable	2% (1)
How comfortable did you feel with IM injections post-training?	
Not at all or somewhat comfortable	24% (12)
Comfortable or very comfortable	76% (38)
How comfortable do you feel with IM injections now [i.e., post vaccination program)?	
Not at all or somewhat comfortable	0% (0)
Comfortable or very comfortable	100% (49)
How comfortable did you feel talking to patients about the COVID-19 vaccine prior to volunteering?	
Not at all or somewhat comfortable	70% (35)
Comfortable or very comfortable	30% (15)
How comfortable do you feel talking to patients about the COVID-19 vaccine after volunteering?	
Not at all or somewhat comfortable	0% (0)
Comfortable or very comfortable	100% (50)
Would you sign up for similar volunteer efforts in the future?	
Yes	100% (50)
No	0% (0)

**Table 4:** Student perceptions of the vaccination program.

Program Evaluation Question
Why did you volunteer? To help counsel patients with vaccine hesitancy and to get reach socioeconomic-disadvantaged populations. Wanted to work with vulnerable communities to help them get access to these life-saving vaccines. To reduce disparity in vaccine access. I felt lucky to be offered to vaccine before most people and I wanted to use my protected status to help the community. As a Latino I felt it was my duty to help out the effort of increasing vaccinations in the NYC Latino communities. Wanted to help with pandemic efforts, but also felt a sense of duty and civic responsibility. Wanted to help out in a direct way. I felt it was important and meaningful. This pandemic was a once-in-a-century event, and I was so full of hope that the vaccine would change the trajectory of the pandemic. I was eager to contribute to the pandemic response in a way appropriate to my level of knowledge and training.
How did this add to your professional development?
It gave me a greater understanding and appreciation for the herculean efforts that go into coordinating and rolling out a large-scale public health effort such as COVID-19 vaccination. It provided me with the opportunity to work with a wide variety of patients from all backgrounds and parts of NYC, as well as work with healthcare providers at all levels. Improved communication with diverse patient populations. It gave me experience with IM injections, discussing vaccines, and inter professional collaboration. I felt empowered. Reminded me of my values that had led me to medicine and taught me the fundamentals of IM injection. It made me more comfortable to be back in a patient facing setting especially as a MD-PhD student. I felt like I was able to act less like a student and more like a healthcare professional--helping me develop skills and toolsets to work in the real world. I realized more than ever how much of a difference it makes to simply listen and give time to our patients—Especially those from underserved and historically exploited backgrounds.
How did this experience add to your medical education?
Exposure to a different patient population of NYC. More comfortable doing IM injections. A very unique opportunity to learn the skills of vaccine administration at a time when there was an urgent need. I learned how to discuss questions that patients had about a brand new vaccine technology. This opportunity was the most rewarding experience I’ve had in medical school and was unlike anything in the standard curriculum. Participating in these vaccination events fostered my longstanding interest in public health into a passion and has had a strong impact on my career direction. This was by far the most meaningful part of my medical school education so far…the lack of formal evaluation encouraged self-reflection and self-evaluation about my own skills, which I found empowering and useful. we rarely get to feel like we are doing something that matters! this did. Confidence and autonomy gained from being independent provider.

## Data Availability

Not applicable.

## Supplementary Materials

The following supporting information can be downloaded at: https://www.mdpi.com/article/10.3390/vaccines10071058/s1, Supplementary File S1: Student Vaccination Feedback Survey.
