# Supplementary material for: Students as Community Vaccinators: Implementation of A Service-Learning COVID-19 Vaccination Program
Source: Vaccines (Basel). 2022 Jun 30;10(7):1058. doi: 10.3390/vaccines10071058 (PMC9324302; doi:10.3390/vaccines10071058)
Supplement: Supplementary file 1 [file vaccines-10-01058-s001.zip › vaccines-1775447-supplementary.pdf]

Student Vaccination Feedback Survey

Please complete this survey based upon your experiences volunteering at COVID-19 vaccination sites.

Thank you!

---

1) About how many vaccination events did you attend and serve as a vaccinator?
   - ◯ 1-5
   - ◯ 5-10
   - ◯ 10-20
   - ◯ >20

2) Did you feel you had adequate training and preparation for participation?
   - ◯ Yes
   - ◯ No

3) Did you feel protected wearing appropriate PPE and adhering to NYS guidelines?
   - ◯ Yes
   - ◯ No

4) Did vaccination site supervisors assist with problem management and provide guidance?
   - ◯ Yes
   - ◯ No

5) How comfortable did you feel with IM injections prior to training?
   - ◯ Not at all comfortable
   - ◯ Somewhat comfortable
   - ◯ Comfortable ◯ Very comfortable

6) How comfortable did you feel with IM injections post-training?
   - ◯ Not at all comfortable
   - ◯ Somewhat comfortable
   - ◯ Comfortable ◯ Very comfortable

7) How comfortable do you feel with IM injections now?
   - ◯ Not at all comfortable
   - ◯ Somewhat comfortable
   - ◯ Comfortable ◯ Very comfortable

8) How comfortable did you feel talking to patients about the COVID-19 vaccine prior to volunteering?
   - ◯ Not at all comfortable
   - ◯ Somewhat comfortable
   - ◯ Comfortable ◯ Very comfortable

9) How comfortable do you feel talking to patients about the COVID-19 vaccine after volunteering?
   - ◯ Not at all comfortable
   - ◯ Somewhat comfortable
   - ◯ Comfortable ◯ Very comfortable

10) Could training be improved for these types of events in any way?

    ____

11) Would you sign up for similar volunteer efforts in the future?
    - ◯ Yes ◯ No

12) Why did you volunteer?

    ____

13) How did participating in this humanitarian effort add to your professional development?

    ____

14) How do you think this experience added to your medical education?

    ____

15) Please share any additional thoughts:

    ____
